# Calibrating Doppler Imaging of Preterm Intracerebral Circulation Using a Microvessel Flow Phantom

**DOI:** 10.3389/fnhum.2014.01068

**Published:** 2015-01-13

**Authors:** Fleur A. Camfferman, Ginette M. Ecury-Goossen, Jhuresy E. La Roche, Nico de Jong, Willem van ’t Leven, Hendrik J. Vos, Martin D. Verweij, Kazem Nasserinejad, Filip Cools, Paul Govaert, Jeroen Dudink

**Affiliations:** ^1^Department of Neonatology, Universitair Ziekenhuis Brussel, Vrije Universiteit Brussel, Brussels, Belgium; ^2^Department of Neonatology, Erasmus Medical Centre, Rotterdam, Netherlands; ^3^Department of Biomedical Engineering, Erasmus Medical Centre, Rotterdam, Netherlands; ^4^Department of Imaging Physics, Delft University of Technology, Delft, Netherlands; ^5^Department of Biostatistics, Erasmus Medical Centre, Rotterdam, Netherlands; ^6^Department of Radiology, Erasmus Medical Centre, Rotterdam, Netherlands

**Keywords:** preterm brain, cerebral circulation, cerebral perfusion, microcirculation, flow phantom, calibration, cerebral blood flow, Doppler

## Abstract

**Introduction:** Preterm infants are born during critical stages of brain development, in which the adaptive capacity of the fetus to extra-uterine environment is limited. Inadequate brain perfusion has been directly linked to preterm brain damage. Advanced high-frequency ultrasound probes and processing algorithms allow visualization of microvessels and depiction of regional variation. To assess whether visualization and flow velocity estimates of preterm cerebral perfusion using Doppler techniques are accurate, we conducted an *in vitro* experiment using a microvessel flow phantom.

**Materials and Methods:** An in-house developed flow phantom containing two microvessels (inner diameter 200 and 700 μm) with attached syringe pumps, filled with blood-mimicking fluid, was used to generate non-pulsatile perfusion of variable flow. Measurements were performed using an Esaote MyLab70 scanner.

**Results:** Microvessel mimicking catheters with velocities as low as 1 cm/s were adequately visualized with a linear ultrasound probe. With a convex probe, velocities <2 cm/s could not be depicted. Within settings, velocity and diameter measurements were highly reproducible [intra-class correlation 0.997 (95% CI 0.996–0.998) and 0.914 (0.864–0.946)]. Overall, mean velocity was overestimated up to threefold, especially in high velocity ranges. Significant differences were seen in velocity measurements when using steer angle correction and in vessel diameter estimation (*p* < 0.05).

**Conclusion:** Visualization of microvessel-size catheters mimicking small brain vessels is feasible. Reproducible velocity and diameter results can be obtained, although important overestimation of the values is observed. Before velocity estimates of microcirculation can find its use in clinical practice, calibration of the ultrasound machine for any specific Doppler purpose is essential. The ultimate goal is to develop a sonographic tool that can be used for objective study of regional perfusion in routine practice.

## Introduction

Central to normal human brain development and function is maintenance of adequate blood flow and oxygenation. This requires complex regulatory mechanisms, which at the early stages of human development exist in a vulnerable equilibrium and are notoriously difficult to monitor. Severe neonatal conditions, such as prematurity, birth trauma, infections, and congenital malformations, often compromise brain perfusion during this critical period of brain development and will grow into an avalanche of neurodevelopmental problems, including cognitive deficits, motor disability, and psychiatric diseases (Sherlock et al., 2005; Volpe, 2009) with ensuing lifelong burdens for the growing individuals and their families, and a major socio-economic impact for the health care system and the whole of society (Petrou et al., 2001).

Brain injury as a complication of preterm birth is directly or indirectly linked to low brain perfusion and oxygenation (Meek et al., 1999; Kissack et al., 2004). Although little is known about the ability of the preterm to regulate cerebral blood flow (CBF) in response to changes in perfusion pressure, there is some evidence that the autoregulatory range is limited compared with that of adults and absent in sick infants (Greisen, 2005). Muscular walls are even absent in fragile medullary channels of the preterm brain, suggesting that some functional tools for autoregulation are not present yet (Kuban and Gilles, 1985). Consequently, extremes of systemic perfusion are transmitted unaltered to brain tissue. It is still a challenge to study neonatal organ and brain–blood perfusion systematically. A reliable, objective, repeatable, safe, and bedside method to characterize neonatal blood perfusion is needed.

Alternative methods to approximate blood flow, like blood pressure, diuresis, heart rate, and limb oxygen saturation are extensively used, but are notoriously poor surrogates of neonatal brain perfusion. Standard techniques for adult brain studies are undesirable or very difficult to apply in the neonate. CBF measured with radioactive Xenon or jugular occlusion plethysmography are disruptive to the infant and cannot be repeated frequently. MRI perfusion scans (such as arterial spin labeling) are not suited for (repeated) monitoring and require transport of often critically ill and clinically unstable infants to the radiology ward (Liem and Greisen, 2010). Now, tissue oxygen delivery can be studied using optical near-infrared spectroscopy (NIRS), a bedside and safe technique, which allows continuous monitoring. However, NIRS is not quantitative and only provides indirect information about blood flow in regions that cannot be precisely pinpointed. Although trends in CBF may be inferred from changes in cerebral oxygenation and/or blood volume, NIRS does not allow a direct measure of CBF (van Bel et al., 2008; Marin and Moore, 2011).

EEG monitoring, like NIRS, is a safe and bedside neuro monitoring tool, which gained renewed interest of clinicians and researchers because of improved hardware and software in recent years. However, changes in EEG signal reflect large neuronal electrophysiological changes in a late and often irreversible stage of brain injury evolution (Hellström-Westas, 2006).

Brain ultrasound is a widely used non-invasive and bedside tool for evaluation of neonatal brain anatomy and detection of brain injury. Color Doppler (CD) has already found a limited application in the analysis of patent ductus arteriosus and birth asphyxia. Indices like pulsatility index and resistance index have been developed and used as proxies for flow (Pezzati et al., 2002; Basu et al., 2014). Recent advances in ultrasound technique have made visualization of small diameter vessels (microcirculation) possible (Macé et al., 2011). Yet, conventional pulsed wave Doppler (PWD) is regularly used for absolute flow velocity measurements. Triplex mode imaging is used in current daily practice, in which flow information is overlaid to an anatomical B-mode image, and absolute flow velocities are measured in the PW Doppler panel (WHO, 2011). Flow information is visualized either in CD or power Doppler (PD). In CD, the color indicates average velocity in the vessel, which is sensitive to the angle of the vessel. In PD, the color indicates the total amount of Doppler energy in the echo from the vessel, which is not sensitive to the angle. The general effect is that PD should be able to detect lower flow velocities and smaller vessels, at the cost of loss of quantitative velocity information. Moreover, the additional method of directional PD gives the PD, but estimates the direction of the flow and colors it subsequently.

Although there is no general consensus on the definition of “microcirculation” in the preterm brain, it is often defined as arteriolar and venular flow. Extrapolating from descriptions of vessel anatomy in fetuses, the diameter of microvessels visualized with Doppler in cerebral white matter of a preterm infant between 24 and 30 weeks is expected to be between 50 and 100 μm (Kuban and Gilles, 1985; Anstrom et al., 2004). The large arteries of the circle of Willis of 24-week-old fetuses have diameters around 400–500 μm (Vasovic et al., 2007). High-frequency linear probes permit visualization of vessels with a diameter below 200 μm. Therefore, visualization of the brain microcirculation of the preterm infant is feasible, making this a potential tool for *in vivo*, safe, bedside, repeatable measurement of CBF. Still, one of the challenges of ultrasound is that the vessel caliber cannot be accurately measured, so that flow cannot be estimated (Kehrer et al., 2003, 2005).

Models using a flow phantom and advanced (power) Doppler technique may be of use to objectively quantify regional flow variation. To mimic preterm brain tissue, this model should depict very small vessel sizes (about 200 μm) and very low flow (<2 cm/s).

As far as we know, no studies are reported to assess the accuracy of current PW, CD, and directional PD techniques in the visualization of microperfusion. Our aim was to evaluate if velocity measurements of microvessels are reproducible and accurate using sensitive modern ultrasound techniques. The second aim was to determine which Doppler technique (color versus PD, type of probe) is best used to visualize these small vessels. To this purpose, we set up an *in vitro* experiment using a microvessel flow phantom designed to mimic preterm cerebral perfusion. We hypothesized that current ultrasound technique is sufficiently accurate to estimate flow in preterm brain microvessels.

## Materials and Methods

### Microvessel flow phantom

**Figure 1:**
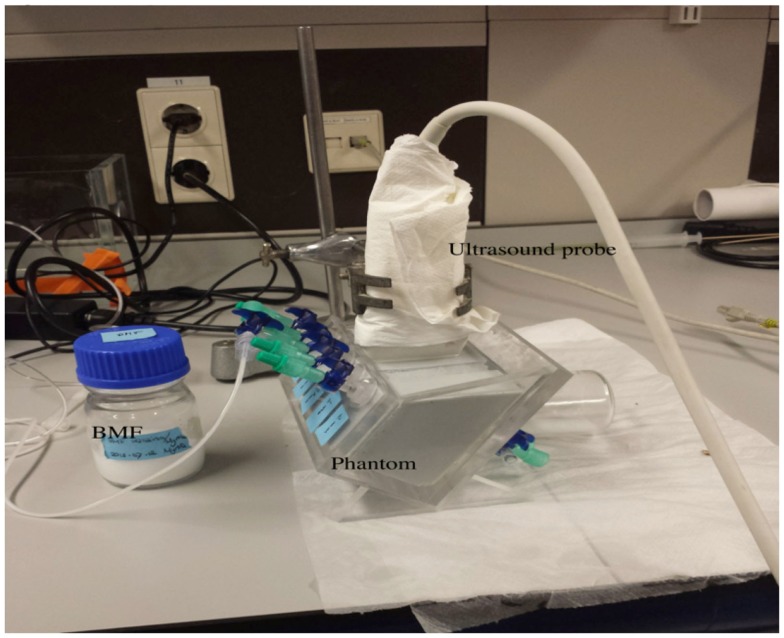
**The test setting**. Flow phantom with a fixed ultrasound probe. BMF, blood-mimicking fluid.

The flow phantom (Figure 1) consisted of an acrylic container filled with agar-based tissue-mimicking material, built according to Teirlinck et al. (1998). The tissue-mimicking material contained two vessels made of polyethylene terephthalate glycol-modified (Paradigm Optics, Vancouver, WA, USA) with an inner diameter of 200 μm each, and three silicone vessels (ERIKS bv, Alkmaar, the Netherlands) with an inner diameter of 700, 1000, and 2000 μm respectively. The blood-mimicking fluid (BMF) used to run through the vessels was based on the recipe by Ramnarine et al. (1998). However, our BMF contains half the amount of dextran and glycerol compared to the original recipe. This reduces the viscosity of the fluid, which is necessary to prevent blockage of the small vessels used in this study.

We intended to simulate the internal cerebral veins (ICV) and intracerebral medullary veins of very preterm infants using this microvessel flow phantom. Based on measurements performed in daily clinical practice, the distance from the anterior fontanel to the ICV is 2.5–5 cm, and intracerebral medullary veins are located at a depth of 2 cm. Blood flow velocity in the ICV is about 5 cm/s and in medullary veins this lies around 1 cm/s (Pfannschmidt and Jorch, 1989; Deeg and Lode, 2005). ICV diameter is estimated to be 500 μm in these neonates, while the diameter of the intracerebral medullary veins is around 100 μm (Kuban and Gilles, 1985). Since capillaries for the flow phantom with a diameter of 100 and 500 μm were not available, we used the 200 and 700 μm vessels for this study.

### Test setting

To generate steady flow through the capillaries, we used a calibrated syringe pump (Harvard Apparatus Pump 11 Elite, Instech Laboratories, Plymouth Meeting, USA) to infuse BMF at velocities ranging from 1 to 10 cm/s. This pump can produce regular flows as low as 1.28 pL/min. To calibrate the syringe pump, we calculated expected BMF volume based on pump speed and duration of the experiment and compared this with the volume of BMF collected at the flow phantom outlet. Recording time was started at the first drop.

For each vessel size, flow settings (microliter per second) were calculated according to the following equation:

(1) $$Q=\left(V_{\mathrm{avg}}\times \pi r^{2}\right)\times 10^{9}$$

*Q* is flow in microliter per second, *V*_avg_ is average velocity in meter per second, and *r* is the radius of the vessel in meter.

Measurements were obtained using an Esaote system (Mylab 70, Genova, Italy) with a linear (Esaote LA 435 Linear Array Ultrasound Probe, 6.0–18.0 MHz) and convex probe (Esaote CA123 Convex Array Ultrasound Probe, 3.3–9.0 MHz). A standard ultrasound preset was used, identical to the preset used for imaging in daily clinical practice. This standard preset was optimized for neonatal preterm microvessel visualization prior to a large local prospective cohort study. The aim of that study was to explore the feasibility of CD for monitoring of cerebral perfusion in very preterm infants at the level of microvessels (unpublished data of Raets et al. – Preterm cerebral microcirculation assessed with color Doppler: a pilot study). According to Ten Cate et al. (2013), the Esaote Ultrasound machine, as used in this experiment, proved to be more suitable for microcirculation imaging in comparison to the other ultrasound brands. This is most probably mainly caused by fundamental differences in processing the Doppler signal or internal settings inaccessible to users. Settings used in this study are summarized in Table 1. For the linear probe, settings included a transmit frequency of 7.7 MHz in flow visualization and 5.9 MHz in PW Doppler, a gain of 50%, and an imaging depth of 30 mm. For the convex probe, settings included a transmit frequency of 5 MHz in flow visualization and 6.3 MHz in PW Doppler, a gain of 54%, and imaging depth of 76 mm. Thermal and mechanical indices were always kept below 1.0.

**Table 1:** **Ultrasound settings used in this study**.

Doppler mode	Probe	Frequency (MHz)	Gain (%)	PRF (kHz)	PRS	PRC	Wall filter
PWD	Linear	5.9	53–60	0.5–10	–	6	100 Hz
	Convex	6.3	50–56	0.75–2	–	6	65 Hz
CD	Linear	7.7	50	0.37	16	M/2	1
	Convex	5	64	1.5	16	H/2	2
Directional PD	Linear	7.7	50	1–2.5	3, 4	M/2, H/2	2, 3
	Convex	5	64	1–2	3, 4, 6	L/2, M/2	2, 4

*PWD, pulsed wave Doppler; CD, color Doppler mode; PD, power Doppler; PRF, pulse repetition frequency; PRS, persistence; PRC, processing*.

The syringe pump was set to generate non-pulsatile perfusion of predefined volumes for each of the vessels (see above). Using the convex and linear probes, peak flow velocity and diameter of the capillaries were measured at 2 cm depth. Detection and diameter sizing measurements were performed using CD and directional PD. To obtain diameter measurements, a Doppler image was frozen, and diameter was estimated using a manual caliber measurement. Velocity measurements were performed in the PW Doppler panel by manually putting a marker at the extreme bound of the PW profile. Since the flow was non-pulsatile, this extreme bound was persistent over the entire measurement period (1 or more seconds) and easily identifiable. When enabled, the steering angle was aligned parallel to the capillary. The velocity measurements were repeated three times at each predefined velocity. In order to minimize bias, a single observer obtained the measurements in the presence of a second observer.

In order to prevent motion artifacts, the probes were fixed using a tripod. With set flow velocities of 5 cm/s and above, a pulsatile flow pattern was witnessed. This disturbance vanished when using a 10 mL low friction glass syringe (GASTIGHT #1010, Hamilton Company, Bonaduz, Switzerland) or a 60 mL plastic syringe instead of a 10 mL plastic syringe. Therefore, for all set flow velocities of 5 cm/s and more we used the glass syringe.

### Statistical analysis

To assess the reproducibility of the velocity measurements and whether velocity measured with the ultrasound corresponded with the actual set velocity, one-way random intra-class correlation (ICC) coefficient was computed with single measures. Based on bootstrap sampling, the 95% confidence interval for ICC coefficient was calculated. To compare the measured diameter with the actual diameter of the capillaries, we used the Student’s one sample *t*-test. Statistically significant was assumed if two-sided *p* value was <0.05. All statistical analyses were performed in R (version 3.1.1; R Foundation for Statistical Computing, Vienna, Austria – http://www.R-project.org).

## Results

### Velocity measurements

**Figure 2:**
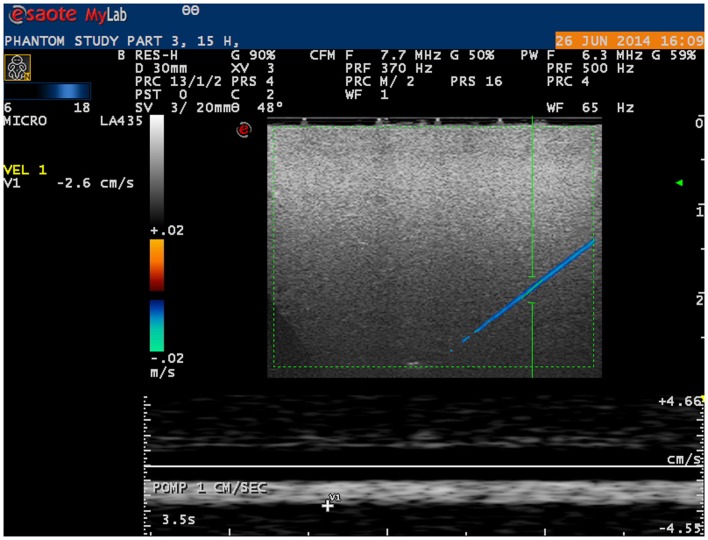
**Ultrasound image of phantom microvessel**. Ultrasound image showing a 200 μm flow phantom catheter as depicted by a linear probe in color Doppler mode.

Microvessels mimicking catheters with a size of 200 and 700 μm with BMF velocities as low as 1 cm/s were adequately visualized using a linear probe (Figure 2). With a convex probe, however, velocities below 2 cm/s in the 700 μm vessel and below 3 cm/s in the 200 μm vessel could not be depicted.

Measurements of velocity and diameter proved to be highly reproducible, with an overall ICC coefficient of 0.997 (95% CI 0.996–0.998) and 0.914 (0.864–0.946), respectively.

As shown in Table 2, in all different settings, ICC coefficient of three consecutive velocity measurements stayed above 0.9 with a variation coefficient below 1.0, except for velocities measured in the 200 μm vessel with the convex probe.

**Table 2:** **Reproducibility of velocity measurements**.

Vessel diameter (μm)	Doppler mode	Probe	Steer angle correction	ICC (95% CI)	
200	Color	Linear	–	0.998 (0.991–1.000)	
			+	0.999 (0.996–0.999)	
		Convex	–	0.985 (0.643–0.989)	^a^
			+	0.991 (0.673–0.995)	^a^
	Power	Linear	–	0.997 (0.991–00.998)	
			+	0.997 (0.988–0.999)	
		Convex	–	0.915 (0.357–0.945)	^a^
			+	0.992 (0.702–0.998)	^a^
700	Color	Linear	–	0.999 (0.996–1.000)	
			+	0.999 (0.976–1.000)	
		Convex	–	0.986 (0.964–0.998)	^b^
			+	0.999 (0.992–1.000)	^b^
	Power	Linear	–	0.995 (0.933–0.997)	
			+	0.999 (0.996–0.999)	
		Convex	–	0.997 (0.991–0.999)	^b^
			+	0.999 (0.993–0.999)	^b^

*Reproducibility of consecutive velocity measurements as showed by ICC (intra-class correlation) coefficients. Color, color Doppler; power, power Doppler; *v*, velocity*.

*^a^No visualization if *v* ≤ 2 cm/s*.

*^b^No visualization if *v* = 1 cm/s*.

**Figure 3:**
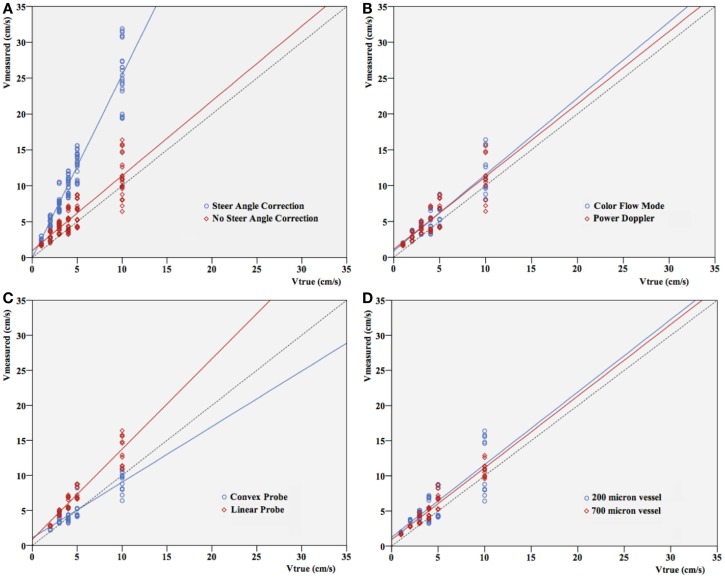
**Pump velocity versus measured Doppler velocity**. Relation between true velocity (centimeter per second) and velocity measured (centimeter per second) in different settings: **(A)** with or without steer angle correction, **(B)** color Doppler versus power Doppler, **(C)** convex versus linear probe, **(D)** vessel size. Since applying steer angle correction leads to significant (*p* < 0.001) overestimation of velocity, velocities in **(B–D)** are without applying steer angle correction. The dotted line represents the line of equality.

Differences between true (pump) velocity and the peak measured Doppler velocity are shown in Figure 3. Overall, PWD overestimated velocity with a 1.1- to 3.5-fold overestimation. This is independent of the flow visualization method in the triplex mode, as expected (Figure 3B). If velocity increased, spreading of the velocities measured in the different settings seemed to be more pronounced. However, this appeared to depend almost uniquely on the highly significant differences that were seen in velocity measurements with versus without steer angle correction (*p* < 0.001). When leaving out the steer angle velocity estimates, correlation between true velocities and measured velocity ameliorated.

**Figure 4:**
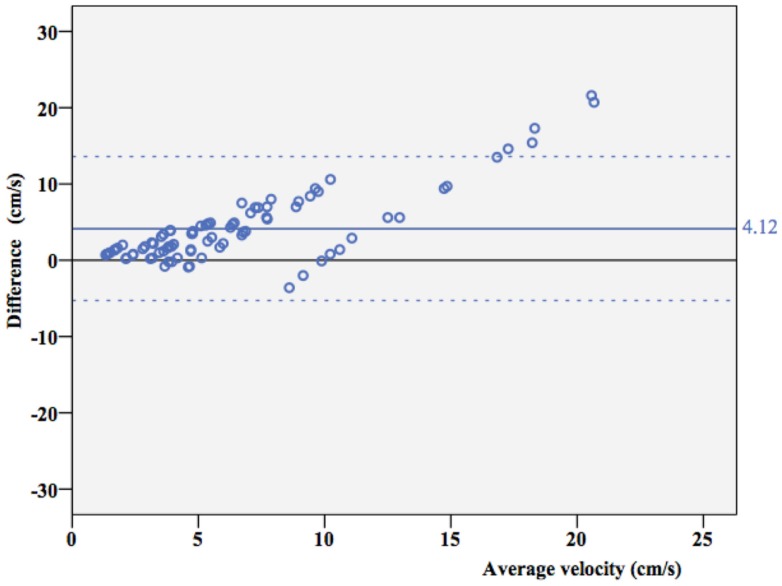
**Agreement between true velocity and Doppler velocity**. Bland–Altman plot of agreement between true velocity (*V*_true_) and velocity estimated by Doppler measurements (*V*_measured_). The mean difference is 4.12 cm/s (95% CI 3.10–5.15 cm/s), dotted lines represent ± 2 SD borders.

Overall, ICC coefficient between average velocity measured by Doppler and the true velocity was 0.352 (0.269–0.442). As shown in a Bland–Altman plot (Figure 4), overall agreement between true velocity (Velocity pump) and velocity estimated by Doppler was poor. However, results suggest consistent overestimation.

### Diameter measurements

**Figure 5:**
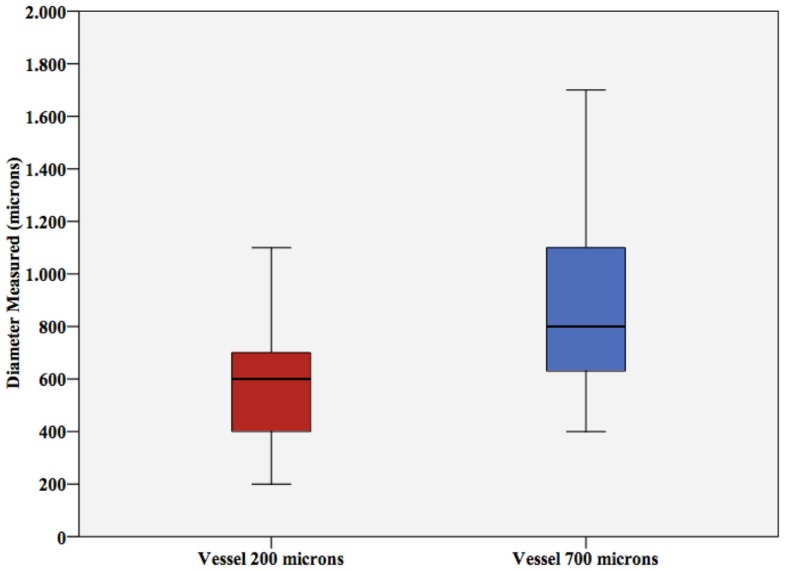
**Vessel diameter**. Vessel diameter measured (±SD) for the 200 μm vessel and the 700 μm vessel. Note the significant overestimation.

Student’s *t*-test for vessel size showed significant differences in diameter estimation (*p*-values <0.001 and 0.005, respectively). As shown in Figure 5, vessel size was mostly overestimated by ultrasound.

## Discussion

In this flow phantom study, we found that Doppler ultrasound imaging is able to visualize microvessel-size catheters with low flow, mimicking cerebral microcirculation. Within the different settings, reproducible velocity and diameter results can be obtained. Our overall results showed that vessel diameter and velocity are overestimated when measured by Doppler ultrasound. This corresponds with results of previous phantom studies (Goertz et al., 2000; Raine-Fenning et al., 2008a; Schulten-Wijman et al., 2008; Xu et al., 2008). However, these studies were designed mainly to determine optimal machine settings and minimal detectable flow. To our best knowledge, this is the first *in vitro* experiment to calibrate for neonatal brain microcirculation.

Overestimation of flow velocity was observed in our study with convex as well as linear ultrasound probes. Previous publications (Guo et al., 1995; Teirlinck et al., 1998; Schulten-Wijman et al., 2008) described a similar phenomenon. Several aspects of Doppler technique and processing of the signals may contribute to this overshooting, as detailed below.

First, it might be explained by sampling inaccuracy. The flow can be assumed parabolic in the low-Reynolds number regime asserted in our measurements. In such a flow, the peak velocity is two times higher than the average flow as predicted by Eq. 1. Moreover, we manually picked the maximum Doppler velocity in the PW panel, as in current clinical practice, not the average velocity. The effect of the flow profile on such Doppler-based velocity estimation is complex, but tends to overestimate the mean velocity (Ricci et al., 2014).

A second explanation might be the way that raw ultrasound data are filtered and processed within the ultrasound machine to produce an image. Most ultrasound machines consider the mean propagation speed *c* in tissue to be 1540 cm/s and use this value to calculate many values, of which velocity is 1 (Zagzebski, 1996; Gill, 2012). If the true propagation speed is higher than 1540 cm/s, this may lead to overestimation of predicted velocity. Exact propagation speed of preterm brain tissue is unknown and additionally depends on several other factors, like temperature and hydration status (Kremkau et al., 1981). Also, in the process of filtering background scatter, some information might be lost or adapted, all according to the software used. At present, we are not familiar with the manufacturer’s confidential post-processing algorithms to create an image from raw data. Especially, when desiring to depict smaller vessels and lower flow velocities, insight in these methods and cooperation with the manufacturer in the development of new software might be of importance.

Third, overshooting might be explained by insonation angle. Since both velocity and direction of blood flow determine Doppler shift, which is the main principle of Doppler ultrasound, insonation angle is an important determinant in calculating velocity. To make velocity quantification error as small as possible, the Doppler angle must be kept as small as possible (Zagzebski, 1996). Current study was performed in a flow phantom with an insonation angle of about 60°, which is considered the maximal insonation angle in clinical practice but still gives an important velocity estimation error. Additionally, increasing insonation angle worsens several Doppler artifacts, such as an intrinsic spectral broadening, which leads to a systematic overestimation in velocity measurements (Gill, 2012).

Pulsed wave Doppler and CD ultrasound depend on the time shift of pulsed wave echoes to calculate velocity and direction. In PD, on the other hand, the color displayed is determined by the Doppler signal power rather than the effective time shift. In theory, this makes PD more sensitive for visualization of low flow velocity (Murphy and Rubin, 1997; Martinoli et al., 1998). Yet, our study shows that both directional PD and CD are able to detect flow velocities down to 1 cm/s with the linear array, which would indicate applicability of both methods in the current framework of preterm intracerebral circulation imaging. The measured velocities with PD were independent of the CD or PD visualization method, which is expected because of the independent nature of data acquisition and processing between PW Doppler and the visualization method.

As expected, applying the steer angle artificially corrects for the angle between flow direction and ultrasound beam. Since the measured Doppler value is divided by the cosine of the angle that the sonographer sets, it should always result in larger apparent velocities in the image (Szabo, 2013). This is consistent with our findings.

Considering the specific characteristics of the linear and convex ultrasound probes and their use in clinical practice, we expected the linear probe to be more suitable for microcirculation imaging. Indeed, lower flow velocities were visualized using the linear probe in the 200 μm vessel compared to the convex probe. As seen in Figure 3, velocities were mainly overestimated by the linear probe while the convex probe underestimated them. Apart from that, results show no obvious superiority for this probe and its specific settings, with reference to the diameter and blood flow velocity of the microvessels.

Since B-mode ultrasound is insufficiently accurate in estimating the diameter of microvessels, we tried to approach true diameter using CD and PD. Reproducible results were obtained; however, overestimation was equally observed as in the velocity measurements. This inaccuracy in measuring diameter is a known problem of Doppler technique. Due to the relatively long transmit pulses in Doppler, axial accuracy of CD systems is rather low, resulting in the encoding of the color sometimes beyond the true flow field (Guo et al., 1995).

Additionally, when adjusting instrument parameters, for instance, increasing the gain, overestimation of vessel diameter is more likely (Browne et al., 2004; Raine-Fenning et al., 2008b). Mitchell (1990) stated that a jet area depicted by color imaging is likely to be more dependent on instrument parameters than on vascular anatomy or physiological characteristics. However, current understanding of ultrasound technique and new *in vitro* models like this microcirculation flow model might give the opportunity to calibrate machine settings for measurement of diameter and true blood flow in near future. In a small spinoff pilot study, ultrasound settings were adjusted according to flow phantom measurements allowing accurate caliber visualization during Doppler imaging, showing the feasibility of this technique.

In conclusion, Doppler ultrasound might be an additional diagnostic tool for quantification of neonatal brain blood flow. This study shows that, before starting to use velocity estimates in patients, calibration of the ultrasound machine for that purpose is needed. The ultimate goal of this type of research is to develop a sonographic tool that can be used for objectively studying regional (3D) perfusion in routine practice.

## Author Contributions

Fleur A. Camfferman has written the manuscript and created figures. Ginette M. Ecury-Goossen has performed the analysis and has written the manuscript. Jhuresy E. La Roche has performed the analysis. Hendrik J. Vos provided technical support and has written the manuscript. Nico de Jong, Willem Van ‘T Leven, and Martin D. Verweij provided technical support and contributed to the manuscript. Kazem Nasserinejad analyzed the data and contributed to the manuscript. Filip Cools contributed to the manuscript and the figures. Paul Govaert and Jeroen Dudink have written the manuscript, designed the experiments, and provided expert consultation. Additionally, Jeroen Dudink provided senior supervision.

## Conflict of Interest Statement

The authors declare that the research was conducted in the absence of any commercial or financial relationships that could be construed as a potential conflict of interest.

## Abbreviations

BMF, blood-mimicking fluid; CBF, cerebral blood flow; CD, color Doppler; ICC, intra-class correlation; ICV, internal cerebral vein; NIRS, near-infrared spectroscopy; PD, power Doppler; PI, pulsatility index; PRC, processing; PRF, pulse repetition frequency; PRS, persistence; PW, pulsed wave; PWD, pulsed wave Doppler; RI, resistance index.
